# Beyond the Bottle: Exploring Health-Promoting Compounds in Wine and Wine-Related Products—Extraction, Detection, Quantification, Aroma Properties, and Terroir Effects

**DOI:** 10.3390/foods12234277

**Published:** 2023-11-27

**Authors:** Catarina Marques, Lia-Tânia Dinis, Maria João Santos, João Mota, Alice Vilela

**Affiliations:** 1 Centre for the Research and Technology of Agro-Environmental and Biological Sciences (CITAB), University of Trás-os-Montes and Alto Douro, P.O. Box 1013, 5001-801 Vila Real, Portugal; catarina.ipsmarques@gmail.com (C.M.); liatdinis@utad.pt (L.-T.D.); 2 University of Trás-os-Montes and Alto Douro, P.O. Box 1013, 5001-801 Vila Real, Portugal; majoao.ssantos@gmail.com (M.J.S.); joao.mota170@gmail.com (J.M.); 3 Chemistry Research Centre (CQ-VR), Department of Agronomy, School of Agrarian and Veterinary Sciences, University of Trás-os-Montes e Alto Douro, P.O. Box 1013, 5001-801 Vila Real, Portugal

**Keywords:** phenolic and non-phenolic compounds, climate, topography, geology, saliva pH and enzymes, sensory perception, vinegar, wine spirit

## Abstract

Health-promoting compounds in wine and wine-related products are important due to their potential benefits to human health. Through an extensive literature review, this study explores the presence of these compounds in wine and wine-related products, examining their relationship with terroir and their impact on the aromatic and flavor properties that are perceived orally: sunlight exposure, rainfall patterns, and soil composition impact grapevines’ synthesis and accumulation of health-promoting compounds. Enzymes, pH, and the oral microbiome are crucial in sensory evaluation and perception of health promotion. Moreover, their analysis of health-promoting compounds in wine and wine-related products relies on considerations such as the specific target compound, selectivity, sensitivity, and the complexity of the matrix.

## 1. Introduction

Recent studies have brought attention to the potential health risks associated with alcohol consumption, including wine [1,2]. While acknowledging these concerns, this study delves into the multifaceted aspects of wine and its related products, exploring wine’s health-promoting compounds and potential risks associated with wine consumption. In contemporary food and beverage industry research, scientific investigations focus on identifying health-promoting compounds inherent in plants or derived materials. Most of these compounds belong to the polyphenol family, encompassing flavonoids and non-flavonoids. Additionally, carotenoids, antioxidants, and essential vitamins and minerals offer discernible health benefits upon consumption [3,4].

Wine and wine-related products are complex matrices containing thousands of these compounds, many with nutraceutical properties. Indeed, the contribution of wine to the Mediterranean diet (MD) has been proved by epidemiological studies [5] and clinical intervention studies [6], which show that it affects longevity and is an optimal diet for the prevention of coronary heart disease and cancer, helping in the reduction of triglycerides, homocysteine, total cholesterol, fibrinogen, blood glucose concentration, and diastolic/systolic blood pressure, while increasing HDL-cholesterol and apoA1 [7,8].

Considering the polyphenols, in wine, the non-flavonoid fraction is characterized by hydroxybenzoic acids (protocatechuic acids, gallic, and syringic), stilbenes (resveratrol and piceatannol), and hydroxycinnamic acids (coumaric and caffeic acids) [9,10]. Concerning flavonoids, wine contains anthocyanins (malvidin), flavonols (quercetin), flavanones, flavones, flavan-3-ols, and proanthocyanidins in their aglycone or glycosylated form [11,12]. Due to the presence of these molecules, red wine is associated with preventing cardiovascular diseases when consumed in moderate quantities. Functional compounds like polyphenols, namely resveratrol, quercetin, and catechins, which have antioxidant, anti-inflammatory, and cardioprotective effects, are at the heart of the matter [13,14]. Furthermore, for the consumer, preventing diseases by altering dietary conduct presents more benefits than medical care, and currently, the nutritional value appears to be the health benefit that has the most impact on a consumer’s purchase [15]. Nevertheless, health-promoting compounds are prevalent throughout the transition from grapes to wine and its related products [16].

Wine production yields a wide range of related products. The primary product, wine, exists in diverse types such as red, white, and rosé, each boasting unique flavor profiles and characteristics. The distinction between white and red wines in wine production lies primarily in the grape skins. For white wines, grapes are pressed, and only the juice is fermented, excluding the skins and seeds. This process retains the grapes’ natural color, producing a clear, pale wine. In contrast, red wines are made by fermenting the juice and the skins together. This prolonged contact with the skins imparts the wine with its characteristic red hue, tannins, and additional flavors, creating a more complex taste profile. Rosé wines, on the other hand, are crafted by allowing brief contact between the grape skins and the juice, resulting in a delightful spectrum of pink tones and a nuanced flavor profile that combines elements of red and white wines.

In addition to wine, the vinification process yields valuable byproducts. Grape pomace, the residual material derived from grape pressing, is frequently used in producing pomace brandy or grape seed oil. Moreover, it serves as a source for various other products, including tartrates, citric acid, hydrocolloids, bioactive compounds, and dietary fiber [17]. Grape skins and seeds, rich in antioxidants, find applications in dietary supplements and cosmetics [18]. Moreover, the remnants of grapes, known as must, are employed in numerous ways, such as producing grape juice concentrates or as a natural sweetener [19]. Additionally, wine production gives rise to wine vinegar, a versatile condiment used in culinary applications, and brandy, a spirit derived from distilled wine.

Like brandy and wine vinegar, wine-related products have numerous human health benefits associated with bioactive compounds. However, the concentration and type of combinations in wine-related products may vary depending on the terroir of the wine base used and the production process. Brandy is associated with increased high-density lipoprotein (HDL) cholesterol, which promotes heart health when consumed moderately [20]. The primary organic acid of wine vinegar is acetic acid, and it has been shown to have antimicrobial and antioxidant properties [21]. It also suppresses body fat accumulation [22]. Wine vinegar from red wine contains bioactive compounds with antimicrobial, antioxidant, antidiabetic, antitumor, and anti-obesity effects. They are associated with reducing the risk of chronic diseases such as cardiovascular disease and cancer [23]. Wine vinegar has considerable potential concerning its influence on human health. However, further research is needed to understand how it may contribute to a healthy diet.

The sensory perception of wine and wine-related products’ health-promoting compounds could be more credible and can be modulated. Sensory evaluation plays a critical role in wines and wine-related products since it evaluates essential attributes such as color, clarity, odor, taste, and astringency, among others, like mouthfeel. However, these sensory characteristics can be modified by the presence of health-promoting compounds, namely polyphenols, and their low solubility [24]. Muñoz-Bernal et al. [25] modified the phenolic profile of wines by adding GPE (grape pomace extracts), mainly the flavanol and flavan-3-ol fractions. The modification of the wine phenolic profile induced two sensory characteristics: astringency and sweetness. Thus, it is possible to modulate the nutritional characteristics of wines and wine-related products and their sensory perception and pleasantness. For all these reasons, extracting, quantifying, and characterizing the health-promoting compounds available in wines and wine-related products is essential.

The choice of an analytical method to identify these types of compounds depends on the combination of several factors, including the compound of interest, the sensitivity (the ability of an analytical approach to detect minor differences in the concentration of an analyte), the selectivity (ability to differentiate and quantify the target analyte without the interference of other compounds present in the sample) of the method, and the complexity of the matrix [26]. The viticultural products present a complexity of compounds present in the solution that may interfere with the analyses [27,28,29,30]. However, to overcome these challenges, some methods involve sample preparation techniques to assist in isolating the target analyte and removing interferences. Overall, analytical methods require specialized or modified techniques to ensure accurate and precise results and allow for the detection and quantification of the analyte under study [27,31]. Thus, the main aim of this study is to provide a detailed review of the essential health-promoting compounds found in wine and wine-related products, considering the influence of vine terroir, chemical detection and quantification, and their sensory perception. Specifically, it aims to identify what definite health-promoting compounds are present in wine and wine-related products, how climate conditions affect the synthesis and accumulation of these compounds in grapevines, and how alterations in the oral microbiome impact the perception of flavors and aromas in wine products.

## 2. Health-Promoting Phenolic Compounds

### 2.1. Flavonoids and Non-Flavonoids

**Figure 1:**
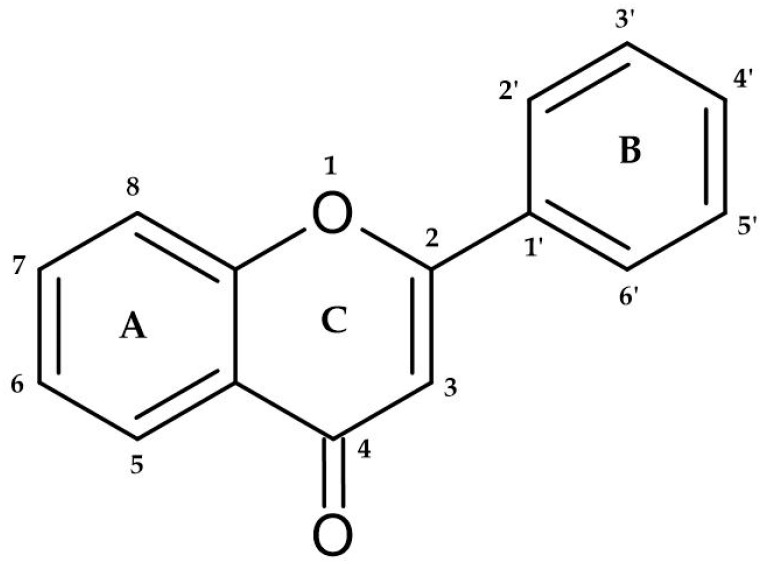
The base structure of flavonoids. The two C6 units denote benzyl rings (rings A and B), and the C3 unit designates the chromane ring (rings C). Depending on the hydroxylation and substitution patterns and the degree of saturation of the chromane ring, flavonoids may exhibit various substituent groups. These substituent groups play a crucial role in determining the specific categorization of each flavonoid corresponding to the labeled numbers.

The phenolic compounds represent a heterogeneous group of chemical compounds widely studied for their human health properties. These compounds can remove free radicals and contribute to preventing cell damage due to their antioxidant properties study [16]. Among phenolic compounds are diverse subclasses of polyphenols, namely phenolic acids, flavonoids, stilbenes, lignans, and tannins [32]. Structurally, these compounds are divided into non-flavonoid and flavonoid phenolic compounds. Non-flavonoid phenolic compounds are composed of phenolic acids and stilbenes. Phenolic acids can be divided into benzoic and cinnamic acids [33]. Flavonoids can be found in the free state or polymerized with other flavonoids, sugars, or non-flavonoids. They possess a C6-C3-C6 base structure (Figure 1) and undergo modifications within the ring, forming the following structural classes: flavonol, flavones, flavanones, and anthocyanins. These compounds are responsible for several wines’ sensory characteristics, such as color [34], mouthfeel sensations (bitterness and astringency) [35], flavor, and aroma [36]. Chemically, they are critical in wine and wine-related products since they interact with wine proteins [37].

Wine flavonoids can be divided into eight subcategories: anthocyanins, flavonols, flavanols, flavones, flavanones, flavanes, flavononols, and chalcones/dihydrochalcones. At the same time, nonflavonoids include phenolic acids, tannins, stilbenes, coumarins, phenyl ethanol derivatives, lignans, and neolignans [38]. Table 1 provides an overview of the main flavonoids and non-flavonoids of wine and wine-derived products.

In wines, we can find the phenolic compounds of grape origin comprising quantitative and qualitative data, which are influenced by variety, yield, berry area, soil, geographic origin, and climate [46]. Besides their importance to human health, they have been described as an essential tool for characterizing commercial wines and can be used in investigations on the geographical origin of wines [47].

Though most wine phenolic compounds originated from grapes, the winemaking method and yeast strain metabolic activity intervein, changing their structure, composition, quantity, and color characteristics [36,48]. For instance, yeast metabolites, such as pyruvic acid and acetaldehyde, are necessary reactants in the biological formation of anthocyanin–flavanol adducts [36,39], vitisins [39], and color-important vinyl phenol pyranoanthocyanins [36,39,49]. *Saccharomyces* yeasts may contribute to changes in wine color by modifying the wine pH due to organic acid metabolism (production or consumption) [48].

Enzymes produced by yeasts, such as pectinase and β-glycosidase, play a role in the winemaking process. Pectinase may affect phenolic extraction from grapes, contributing to the breakdown of pectins that bind phenolic compounds. Additionally, β-glycosidase can hydrolyze the glycosidic bond of certain phenolic compounds, releasing aglycones and influencing the sensory attributes of the wine [50,51].

Moreover, cell walls may autolyze during yeast fermentation, releasing compounds like mannoproteins. These mannoproteins can interact with wine phenolic compounds, promoting binding interactions and, in some cases, precipitation. While not all phenolic compounds have glycosidic bonds, those that do, such as anthocyanins, can be affected by β-glycosidase activity. This enzymatic action releases aglycones, contributing to the final wine product’s color intensity and flavor nuances [52].

### 2.2. Wine Compounds Produced by Fermentation

#### 2.2.1. Tyrosol, Hydroxytyrosol, and Tryptophol

Bioactive products from microbial metabolisms, like tyrosol, hydroxytyrosol, and tryptophol, are natural phenolic compounds in some foods and beverages, especially wine and wine-related products [53]. The formation of tyrosol, hydroxytyrosol, and tryptophol can be influenced by external factors such as temperature, alcoholic degree, and amino acid concentration [54].

These compounds are recognized for their positive health effects, particularly as antiatherogenic, anticancer, neuroprotective, antidiabetic, lipid-regulating, and anti-obesity, and in promoting cardiovascular health-related benefits [55]. Their antimicrobial and antiviral were also investigated, particularly against COVID-19 effects, as hydroxytyrosol promotes mitochondrial function through activating mitophagy [56]; improving the mitochondria metabolism can reduce the severity of SARCS-COV-2 effects on hepatocytes’ mitochondria, ameliorating COVID-induced liver injury [57].

These compounds are considered health-promoting compounds, especially tyrosol, which can represent a natural product supplement to prevent or treat diarrhea, as it relieves diarrhea in mice by inhibiting *Escherichia coli*-induced activation of the NF-κB pathway in mice [58]. According to Gabbia and coworkers [59], tyrosol can also be considered a novel strategy to counteract hepatic steatosis, fibrosis, and inflammation associated with nonalcoholic steatohepatitis development by modulating the recruitment of immune cells in the liver.

**Figure 2:**
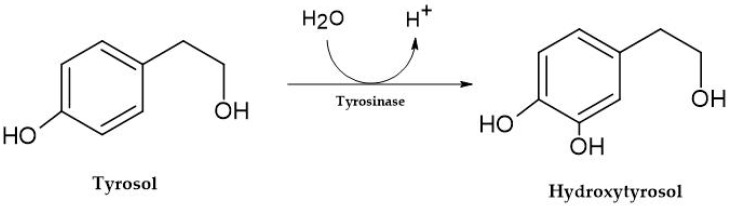
Biosynthesis of hydroxytyrosol.

After ingestion, tyrosol is partially biotransformed into hydroxytyrosol (Figure 2) [60]. The concentration of tyrosol in wine can range from 20 to 60 mg/L in red wines and up to 45 mg/L in white wines [61]. The exact concentration can vary widely among different wine varieties and styles. For example, certain red wines, such as Cabernet Sauvignon, may contain higher tyrosol due to the independent and interactive effects of Cabernet Sauvignon plant materials implanted in different geographical locations [62].

Although olive oil is the primary source of hydroxytyrosol from the Oleuropein degradation by the action of a β-glucosidase and posterior hydrolysis in the human diet, wine is another crucial source due mainly to the yeast’s metabolism [47]. Several studies consider concentrations from 3.66 mg/L to 4.20 mg/L in red wines and 1.72 mg/L to 1.92 mg/L in white wines [63]. This bioactive compound has uncounted health-promoting properties, namely antioxidant, cardiovascular, antidiabetic, and neuroprotective effects [63]. Considering tryptophol in wine, the concentrations found in red wine range from 11.20 mg/L to 24.77 mg/L and in white wine from 4.90 mg/L to 11.26 mg/L, depending on variety [64] and fermentation parameters, namely temperature, alcoholic degree, and amino acids concentration [54]. Tryptophol shares a structural kinship with tyrosol and hydroxytyrosol as constituents of the aromatic alcohol class. Tryptophol is derived explicitly from tryptophan during the fermentation of wine. At the same time, tyrosol and hydroxytyrosol are commonly found in olive oil, and their presence in wine is typically associated with aging in wooden barrels or contact with grape skins [53,54,63].

#### 2.2.2. Melatonin and Serotonin

Bioactive compounds like melatonin and serotonin have incredible health benefits. Melatonin (MEL) is a neurohormone (n-acetyl-5-methoxytyramine) from the pineal gland produced as a secondary metabolite in the plant kingdom. It is produced by O-serotonin methylation followed by n-methoxy tryptamine acetylation in yeast [53] and can also be synthesized from tryptophan, 5-hydroxytryptophan, serotonin, and ultimately n-acetyl serotonin. This molecule is a powerful antioxidant and is very efficient in re-establishing the circadian rhythm, helping in sleeping disorders. It successfully protects against neurodegenerative diseases like Parkinson’s, Alzheimer’s, Huntington’s, amyotrophic lateral sclerosis, and fibrinogenesis [65].

Serotonin is formed during malolactic and alcoholic fermentation by the action of yeast and lactic acid bacteria, and it is derived from the decarboxylation of L-tryptophan [66]. According to Sagonas et al. [67], serotonin is a crucial mediator in fibrosis and vasculopathy, helping in diseases like systemic sclerosis.

**Figure 3:**
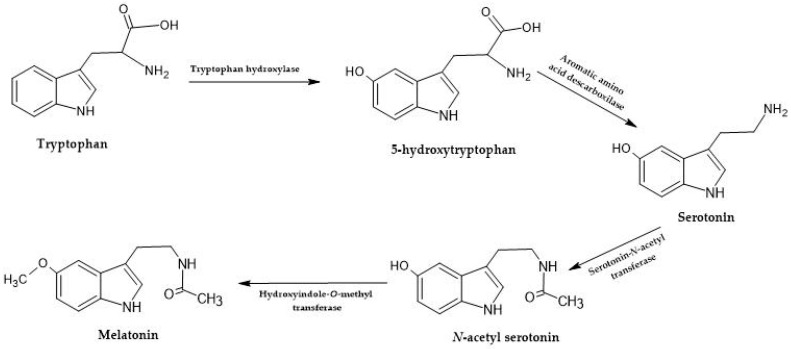
Tryptophan is synthesized into melatonin and serotonin through a series of enzymatic steps. Tryptophan hydroxylase is the first enzyme involved, and it adds a hydroxyl group (-OH) to tryptophan, forming 5-hydroxytryptophan (5-HTP), with the help of tetrahydrobiopterin (BH4). The following enzyme, aromatic L-amino acid decarboxylase (AADC), then removes the carboxyl group (-COOH) from 5-HTP, resulting in the formation of serotonin (5-hydroxytryptamine). To produce melatonin, serotonin is subjected to two enzymatic steps. First, serotonin N-acetyltransferase (SNAT) transfers an acetyl group (-COCH_3_) from acetyl coenzyme A (acetyl-CoA) to serotonin, forming N-acetyl serotonin. Then, N-acetyl serotonin methyltransferase (ASMT) catalyzes the methylation of N-acetyl serotonin using S-adenosyl methionine (SAM) as a methyl donor, resulting in the production of melatonin [53].

Tryptophan-derived molecules, such as melatonin and serotonin (Figure 3), are present in wine at deficient concentrations, varying from picograms to ng/mL [65]. Concentrations seem low; however, these small amounts are enough in dietary intake to measure their effects by different methods [65]. These molecules have been referenced in wine, and the role of yeasts in their formation is evident [53,68]. However, there needs to be more studies regarding identifying and quantifying these molecules in some wine-related products.

## 3. Health-Promoting Non-Phenolic Compounds

### 3.1. Glutathione

Due to health concerns surrounding the use of SO_2_, already described since the 1980s by Freedman [69] as an inducer of asthma when inhaled or ingested by sensitive subjects, even in high dilution, more attention has been given to alternative antioxidants. Ascorbic acid (AA), glutathione (GSH), and GSH-enriched commercial products are examples of alternatives. They can be added to wines and grape must, and their activity may continue throughout the vinification process [70]. However, glutathione addition is regulated by the Organisation Internationale de la Vigne et du Vin (OIV) with a limited dose of no more than 20 mg/L in the must [71]. This regulation underscores the careful consideration and adherence to international standards regarding using specific additives in winemaking processes, considering variations in regulatory frameworks across different countries. While the European Chemicals Agency (ECHA) and European Food Safety Authority (EFSA) [72,73] report no associated hazards for ascorbic acid, the OIV establishes specific maximum acceptable limits for wine treatment (250 mg/L) and residue in wine (300 mg/L) [74]. Additionally, some countries set their maximum acceptable limits, reflecting variations in regulatory standards. Despite the recommended daily dose for adults being 70 to 150 mg, these divergent limits underscore the importance of considering context-specific guidelines for ascorbic acid use [75].

**Figure 4:**
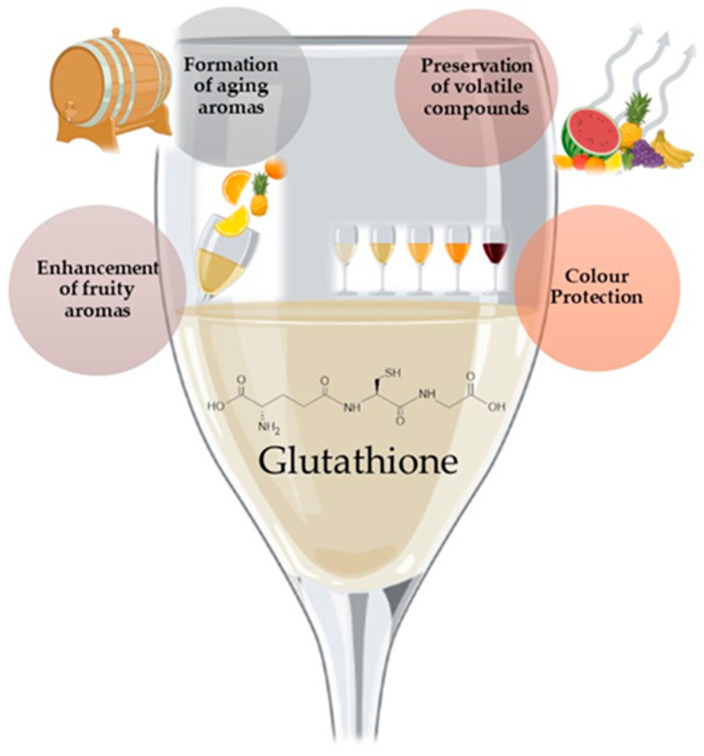
Schematic effect of glutathione in wine and wine-related products.

Glutathione is a thiol-containing tripeptide of glutamic acid, cysteine, and glycine found in many fruits, vegetables, and beverages, including wine [76]. The natural glutathione levels in musts and wine are widely diverse, ranging from non-detectable to 100 mg/L and in wine from non-detectable to 70 mg/L [77]. Glutathione is a powerful antioxidant in wine, helping protect it from oxidative damage. It can directly scavenge free radicals and reactive oxygen species (ROS), which can cause undesirable changes in the wine’s flavor and aroma [78]. Glutathione reacts with aldehydes, like acetaldehyde, responsible for off-flavors in wine and converts them into more stable forms, limiting their accumulation in wine during storage [79]. Additionally, during bottle aging, glutathione protects varietal thiols from oxidation [80]. At the same time, its cysteinyl form can be used as a source of sulfur, increasing the concentration of polyfunctional mercaptans by reacting with trans-2-hexenal to form Glut-3MHal [81]. This process helps to enhance the wine’s aroma, assisting the preservation of compounds produced by yeasts, like esters, such as isoamyl acetate and ethyl hexanoate, terpenes (linalool, α-terpineol), and volatile thiols responsible for wine’s fruity characteristics [82] (Figure 4).

Besides enhancing wine’s fruity aroma, it also helps form aging aromas [77] (Figure 4). In the case of dry Muscat wine, Papadopoulou and coworkers [83] added 20 mg/L of glutathione, inhibiting the vanishing of linalool and α-terpineol during storage of the wine.

Glutathione also contributes to the stabilization of wine’s color intensity and stability. It can limit the formation of browning pigments by trapping o-quinones in a colorless form and restrict the production of xanthylium cation pigment precursors and o-quinone-derived phenolic compounds [80,84].

In humans, glutathione has proven to have antiaging and anti-melanogenic effects [85], and, more recently, a derived form of glutathione (ψ-GSH) can inhibit oxidative stress and neuroinflammation in an Alzheimer’s disease mouse model [86]. Chen et al. [87] studied the effects of glutathione in improving testicular spermatogenesis by inhibiting oxidative stress, mitochondrial damage, and apoptosis induced by copper deposition in mice with Wilson disease.

### 3.2. Vitamins

Vitamins are essential micronutrients that play a vital role in maintaining human health. Vitamin A, a fat-soluble vitamin, is present in wine and wine byproducts such as grape seeds and skins. Studies have shown that vitamin A may help prevent age-related macular degeneration and improve skin health [88].

Additionally, vitamin C is an essential vitamin with antioxidant properties that help protect cells from damage caused by free radicals. Vitamin C is critical for collagen synthesis and immune function [89], and it exhibits antioxidant activities by inhibiting lipid peroxidation and oxidative stress [90].

Vitamin E is another vitamin found in grape seeds and skins. This vitamin has been shown to have antioxidant properties that may help protect against chronic diseases such as cardiovascular disease and cancer [91].

The specific literature on vitamin concentration is scarce. Evers and coworkers [92] referenced an extensive overview of studies on water-soluble vitamins and their concentrations in grapes, grape musts, and wines. Considering this gap in the literature, Evers and coworkers [93] proposed a rapid and reliable method for analyzing vitamins in grape musts. Table 2 compares the water-soluble vitamin content mentioned by Evers et al. (2021) [92] with the concentrations found in the study by Evers et al. (2023) [93].

Although the involvement of vitamins in the development of wine aromas remains primarily unexplored, it is possible to establish some metabolic connections existing between vitamins and aromatic molecules, namely the case of pyridoxine and niacin metabolisms that have been proven to be directly linked to butanoate metabolism, a subsequent essential wine aromatic compounds derived from it, like ethyl butanoate and ethyl 2-methyl butanoate, which are odorant compounds of several wines [92].

### 3.3. Minerals

Several minerals are present in wine and wine-related products, and the most abundant in wine are K, Ca, Na, and Mg. The highest concentration levels can be found in K, between 500 and 1500 mg/L, followed by Ca, Mg, and Na, around 10–200 mg/L. The elements usually present in concentrations ranging from 0.1 to 10 mg/L are Al, B, Cu, Fe, Mn, Rb, Sr, and Zn. Then, ultra-trace elements below 0.1 mg/L, such as Se, Pb, and Cd [94]. These minerals play a crucial role in several physiological processes in the body and have been linked to various health benefits. Calcium is a renowned mineral for its help in bone health and muscle function and can mainly be found in grape skin at levels that can reach 0.8 mg/g fresh weight [95]. While potassium is vital for maintaining fluid and electrolyte balance and regulating blood pressure [96], magnesium has been associated with reduced risk of type 2 diabetes mellitus [97,98] and cardiovascular mortality [99].

While these concentrations may not be exceptionally high compared to other food sources, regular consumption of moderate amounts of wine and wine-related products can contribute to overall mineral intake and provide health benefits. Average wine consumption may be associated as a human health promoter, partly due to the minerals present; however, excessive intake of these minerals can harm human health [94]. It is crucial to note that some metals in these products, such as Pb and Cd, are toxic and unhealthy for human consumption. To safeguard public health, regulations require the analysis of metal content and permissible concentrations in wine. Furthermore, compliance with diverse health protection regulations in different countries necessitates the identification of potentially hazardous substances. These include Cd, Pb, Hg, Al, Tl, As, Sb, S, and several organometallic compounds of Pb and As. [100,101].

In addition, minerals were also detected in wine-related products. In wine vinegar, Paneque and coworkers [102] determined 21 minerals (Al, As, B, Ba, Ca, Cd, Co, Cr, Cu, Fe, K, Mg, Mn, Na, Ni, P, Pb, S, Sr, V, and Zn) in Andalusian wine vinegar by an inductively coupled plasma–optical emission spectroscopy (ICP-OES) method.

The concentrations of minerals in wine and wine-related products can vary depending on several factors, including the grape maturity, variety, soil type, climatic conditions [103], and winemaking process; therefore, the concentrations of minerals can consequently be used to discriminate the geographic origin of wines [104].

## 4. Terroir and Its Impact on Health-Promoting Compounds Synthesis

The concept of terroir holds significant importance in viticulture as it connects the wine sensory characteristics to the environmental factors present during grape cultivation [105]. Terroir is an integrated ecosystem in a specific area, comprising climate, soil composition, topography, the chosen vine rootstock, cultivar [98], landscape features, and biodiversity. Human influences like viticultural practices and oenological techniques can also contribute to the terroir concept [106].

The terroir is challenging to study due to the complex and interactive nature of its various components. The climate, in conjunction with the grapevine variety, tends to exert the most considerable influence at a regional or national level. Within a specific region, geology, geomorphology, and associated topo-climatic effects can play a pivotal role in explaining variations in vine growth and grape characteristics. Some researchers argue that terroir can only be effectively examined in small, carefully mapped areas on a larger scale, particularly when considering soil effects [107,108]. However, this may not necessarily hold for other terroir-related factors such as climate, topography, and geology.

Variations in the climate patterns significantly influence the quality of grapes and, consequently, the resulting wines and their related products. Different grape-growing regions, such as Burgundy in France, Mendoza in Argentina, Douro in Portugal, La Mancha in Spain, and Northeast China, and various regions in Italy, including Tuscany, Piedmont, and Veneto, boast unique climatic conditions impacting grape development, wine characteristics, and typicity. In Burgundy, located in central France, the continental climate with short summers and cold winters results in grapes with relatively low acidity due to milder temperatures during their growth period [109]. Meanwhile, Mendoza in Argentina experiences warm summers and cool nights, fostering Malbec grapes known for their deep color and smooth texture [110]. The Douro region in Portugal features a Mediterranean-like climate with hot and dry summers and mild winters, fostering the growth of renowned grape varieties such as Touriga Nacional and Touriga Franca. The renowned Port wines from the Douro region seem to favor the accumulation of anthocyanidins from exposure to higher altitudes, which show lower temperatures and relative humidity during the last month of maturation [111]. Despite the extreme heat and minimal rainfall, La Mancha in Spain cultivates the Airen grape variety, which thrives in these challenging conditions, producing crisp and light wines [112]. In Northeast China, the temperate humid climate, with temperatures dropping drastically during winter, allows grapes to freeze, enhancing flavor components like sugar and phenols and making them ideal for ice wine production [113]. In Tuscany, the interplay of a Mediterranean climate, diverse soils ranging from clay to limestone, and the cultivation of Sangiovese and other grape varieties yields wines with a distinctive terroir [114,115,116]. In Piedmont, a continental climate, calcareous marl soils, and the cultivation of Nebbiolo, Barbera, and Dolcetto contribute to the renowned character of Barolo and Barbaresco [114,117]. Meanwhile, in Veneto, the diverse climate influenced by the Adriatic Sea, varied soils from alluvial plains to volcanic areas, and the cultivation of Glera, Corvina, Rondinella, and Molinara define the terroir that gives rise to Prosecco, Amarone, and Valpolicella [114,118,119].

The climate of a wine-growing region can affect the accumulation of phenolic compounds, such as stilbenoids and flavonoids, in grapes. Resveratrol (a stilbenoid) accumulation was observed under abiotic stressors such as UV light [120]. Under stress conditions, such as downy mildew (*Plasmopara viticola*) infection, UV light, and AlCl_3_ treatment, the enzyme *o*-methyltransferase is activated, which catalyzes the biosynthesis of pterostilbene, a methoxy of resveratrol [121]. These compounds are known for their antioxidant properties and are associated with potential health benefits, including cardiovascular health [122].

Furthermore, the biodiversity in and around vineyards can promote a healthier ecosystem, indirectly impacting the composition of grapes and wine [123]. Biodiversity can contribute to natural pest control, reducing the need for chemical interventions and fostering a more sustainable vineyard environment [124]. Biodiversity, in turn, can influence the overall quality and composition of the grapes, potentially leading to the formation of compounds associated with health benefits.

The composition of berries is primarily influenced by the climate, specifically the components related to acidity. However, the soil type primarily affects the berry nitrogen, sugar levels, and total anthocyanin content. On the other hand, the vines’ mineral status is mainly determined by the soil type [125].

Lighting also plays a crucial role in grape development and quality. Adequate sunlight exposure promotes the accumulation of desirable compounds. Techniques like leaf removal and reflective mulch enhance grape quality by increasing anthocyanins and flavonoid concentrations [126,127].

Soil quality significantly impacts grape growth and overall wine and wine-related product quality. Influenced by texture and water–fertilizer management, soil quality affects grapevine nutrient absorption and metabolism [128]. Regulated deficit irrigation methods optimize water usage, enhancing grape quality by increasing sugar levels, total phenols, terpenes, and anthocyanins while reducing acidity [129].

Fertilization methods with specific nutrients like nitrogen and calcium impact grape and wine properties [130,131]. Nitrogen is a crucial nutrient in various crops, including grapevines. The nitrogen supply significantly affects vine vigor, crop yield, and berry size and profoundly impacts the significant metabolites of grapes, such as sugar and organic acids. Moreover, nitrogen availability directly influences the production of secondary metabolites, including phenolic compounds, aromas, and aroma precursors in grapes [132]. Trégoat et al. [133] state that berries’ size and malic acid content are restricted by low nitrogen supply. For instance, nitrogen is crucial in synthesizing volatile thiol precursors in Sauvignon Blanc grapes. These volatile thiols are major aroma compounds in various grapevine varieties and are generally associated with a higher quality of wine.

Nitrogen also stimulates the production of glutathione, a compound that helps preserve aroma compounds in musts and wines [134]. This chemical element limits the production of tannins, which can lead to the degradation of volatile thiols [135,136]. Therefore, a moderate nitrogen supply to the vines is desired in white wine production, especially for grape varieties that rely on volatile thiols for their distinctive aromatic profile. However, it is crucial to avoid excessive nitrogen supply, as it can increase the susceptibility of grapes to grey rot caused by *Botrytis cinerea* [137]. Notably, the optimal nitrogen supply differs between red and white wine production, contributing to the variation in soil preferences to produce high-quality white and red wines [125].

Soil composition, particularly the presence of minerals and trace elements, can also contribute to forming phenolic and non-phenolic health-promoting compounds. Evidence suggests that under low-temperature conditions and in the presence of plant stress factors such as fungal infection, there is an observed augmentation in the levels of polyphenolic compounds, particularly resveratrol [89,121,138]. Furthermore, certain nutrient-rich soils, including those enriched with elements like selenium or zinc, have been shown to notably influence the synthesis of compounds possessing valuable antioxidant and anti-inflammatory attributes, such as resveratrol and other phenolic compounds [139]. Also, the shading induces different concentrations of phenolic compounds in the fruits [140], which leads to an additional flavonoid content in wine [141,142]. The soil anchors the vine but supplies vines with minerals, water, and a specific temperature regime in the root zone. Although deep roots can absorb trace elements, no evidence or demonstration can explain how these elements can be converted into aroma compounds or other sensory attributes in wines [143,144]. However, some studies showed that excess soil potassium availability could increase pH in must and wines [145,146]. These factors collectively contribute to the diverse array of worldwide wines, each region producing unique offerings.

## 5. Sensory Perception of Health-Promoting Compounds

### 5.1. Health-Promoting Compound’s Terroir-Dependent Sensory Attributes

Terroir plays a significant role in shaping the wine flavor profile. The unique combination of climate, soil, topography, and biodiversity in a specific wine-growing region contributes to the distinctiveness and complexity of wines produced from that terroir [147]. The climate of a particular wine-growing region dramatically influences the flavor characteristics of the grapes. Cool temperatures make wines with higher acidity and lighter fruit flavors, while temperatures yield riper, fuller-bodied wines with bolder fruit flavors [148]. The composition and structure of the soil impact the mineral content and nutrient availability of the grapevines. Different soils contribute distinct flavors and textures to the grapes, which can be reflected in the wine [125,147]. For example, volcanic soils may impart a smoky or mineral note, while limestone soils can add a chalky or flinty character [149]. The topography of a vineyard, such as its altitude, slope, and exposure to sunlight, influences factors like drainage, sun exposure, and airflow. These factors can affect grape ripening, acidity, and flavor development. For instance, grapes grown on a steep slope might experience better sun exposure, resulting in riper fruit flavors [150], whereas insolation of grape bunches followed by reillumination enhances the flavor of aromatic grape cultivars. This hypothesis was tested by combining gene expression and metabolic analysis of the monoterpene and flavonol synthesis pathways in *Vitis vinifera* L. cv. Riesling [151]. The presence of diverse flora and fauna in and around vineyards can contribute to the overall health and balance of the ecosystem. This biodiversity can indirectly impact wine flavor by promoting natural pest control, improving soil health, and influencing the vineyard environment [152].

Descriptive analysis (DA) is commonly employed to provide a detailed profile of wine’s sensory characteristics [153]. However, it can also serve the purpose of characterizing the typical qualities of a particular wine [154]. A study by King et al. [155] exemplified this by utilizing DA to represent Malbec wines from various vineyard sites in California and Mendoza. This inter-country study revealed that variations in altitude can impact the sensory attributes of a wine, highlighting its role as a factor in terroir and regional typicity. Another survey by Geffroy et al. [156] employed a similar approach and identified the characteristic peppery notes and higher rotundone concentrations in French Gamay wines originating from vineyards in cooler climate regions. In contrast, Kustos et al. [157] explored the regional typicity of Australian Chardonnay and Shiraz wines. They concluded that regional differences alone were insufficient to fully describe the sensory distinctions of the samples, emphasizing the need to consider winemaking elements as well.

### 5.2. Influence of pH, Saliva (Enzymes) Biochemistry, and Oral Microflora on the Sensory Perception of Health-Promoting Compounds

Investigating the factors influencing flavor perception can provide insight into promoting healthy food choices and maintaining good nutrition. In addition to food and beverage characteristics, oral physiology, specifically the saliva [158] and microbiome [159], significantly shapes flavor perception.

Considered the “mirror of the body,” saliva is a complex biological fluid of enormous importance for humans [160,161]. The constitution of saliva is based on approximately 99% water and the remainder in inorganic and organic compounds. The former includes sodium, potassium, calcium, magnesium, chlorides, and carbonates. Regarding the organic compounds, these encompass enzymes—for example, α-amylase, lipase, lysozyme, and esterases—and various proteins, such as immunoglobulins, mucins (glycoproteins), proline-rich proteins, and hormones, among others [162,163,164]. According to recent data [165], the pH of saliva is between 6.2 and 7.4. Slightly acidic or basic, saliva is a highly viscoelastic fluid. It has unique properties that allow the performance of numerous functions, such as lubrication, wetting, emulsification, and changes in surface tension to prevent friction between particles and oral surfaces [166,167]. The above properties also facilitate chewing, digestion, homeostasis, and taste perception and even have antimicrobial properties and buffering capacity [160,166,167]. Like other bodily fluids, saliva has a buffering ability to absorb or release hydrogen ions (H^+^) to regulate their concentration changes and stabilize the pH value [168,169]. A study conducted in 2022 [170], which studied the influence of solutions (aqueous and hydroalcoholic) and drinks, found that saliva influences the pH change. In this study, there were differences in the pH values according to the beverage analyzed; in the case of red and white wines, the pH decreased after contact with the taster’s saliva. This result may be related to the fact that the wines are composed of different acids and in different concentrations. Tartaric, malic, lactic, and citric acids, among others present in wine, may regulate the wine’s pH and confer a buffering capacity [168]. Depending on the acid components present, they may also inhibit the effect of saliva on pH.

Salivary proteins are crucial in the oral cavity, where the various components of saliva interact to create new compounds. These proteins perform multiple functions such as oral digestion (amylases and lipases), detoxification (proline-rich proteins and statins), defense against microorganisms (immunoglobulins and peroxidases), lubrication of the oral cavity (mucins), and the transportation of taste molecules (lipocalins) [170].

An essential protein found in human saliva is α-amylase, which is involved in the breakdown of carbohydrates and starches during the digestive process [171,172,173]. This enzyme is an endoglycohydrolase encoded by the Amy1 gene. It hydrolyses internal α-1,4-glucoside bonds of starch to the disaccharide maltose and moderate-length oligosaccharides called boundary dextrins. Amylase is inactivated at the acidic pH of the gastric lumen. Still, it is more stable in the presence of the pH of oral saliva or in solutions where the pH is closer to the natural pH of saliva—between 6.2 and 7.4 [165]. A study carried out in 2023 [170] reinforces the previous statement, since in aqueous and hydroalcoholic sucrose solutions tested—where the pH was between 5.67 and 6.19, respectively—there was a high activity of α-amylase, showing that this is protected when in contact with solutions and capable of assisting in the digestion of carbohydrates and starch.

Wine has numerous compounds in its constitution that can influence amylase activity. Within the study by Santos and coworkers [170], red wine, generally considered fruity and sweeter, showed higher activity of this enzyme than white wine. The compounds responsible for the enzyme activity are directly related to the sweetness of the wine and are mainly sugars (glucose and fructose), glycols, and glycerol. Moreover, some wines may contain residual sugars that contribute even more to the sweetness of fortified wines. On the other hand, there are compounds capable of inhibiting the activity of amylase, as is the case of tannins. These can bind to proteins, including enzymes, and form stable complexes. Thus, tannins are believed to inhibit α-amylase activity [170]. Regarding aromatic compounds, they tend to be adsorbed by the mucosal film. However, the presence of tannins and their aggregation with the mucosal film could lead to disturbances in the interaction with the aromatic compounds [174].

Variation in the activity and concentration of the α-amylase enzyme is linked to stress, making it a valuable marker for predicting stress levels, whether physical or psychological [143,152,175]. The secretion of the enzyme by the salivary glands is directly related to the activation of the autonomic nervous system [151,152,154]. Therefore, it is safe to say that the activity and concentration of this enzyme depend on the degree of stress to which the individual is subjected during the saliva collection process [154,164,173]. Furthermore, the methods used to determine and interpret the behavior of the α-amylase enzyme must also be considered and adapted to this study’s objective.

The enzyme lipase catalyzes the hydrolysis of ester bonds in the structure of triglycerides (lipids) and releases monoglycerides and free fatty acids [171,176,177]. Lipids are the main constituents of fat; it is possible to state that lipase is one of the main factors influencing the taste perception of fat, impacting its aroma and taste [163,176]. The concentration of the lipase enzyme in human saliva is low [171], and its activity is also related to the composition of the beverages being tasted [170]. Working with complex drinks that contain triglycerides and fatty acids in their composition, coming from both the raw material (grapes, wine) and the fermentative activity of yeast [178,179], will lead to more significant action of this enzyme. Regarding aroma, the concentration of short-chain fatty acids, especially the volatile fatty acids isovaleric and butyric, exceeding the threshold of sensory detection, can form an unpleasant smell (such as cheese or sweaty feet aroma) in beer and wine, thus being considered a defect often of microbiological origin [180].

Another crucial factor in taste perception, apart from the influence of saliva and its constituents, is the oral microbiota composition [159,165]. The human mouth hosts hundreds of bacterial species and, according to the Human Oral Microbiome Database (HOMD), contains over 750 species of prokaryotes. The bacterial diversity varies between different regions of the oral cavity, with the saliva and tongue microbiome showing the most outstanding richness, dominated by the genera *Rothia*, *Prevotella*, *Streptococcus*, *Veillonella*, *Fusobacterium*, *Neisseria*, and *Haemophilus* [181].

Two dominating mechanisms have been suggested that support the role of the oral microbiota in taste sensitivity: (i) Biofilm formation that can limit the access of tastants to their receptors; (ii) microorganisms metabolic activity that can mediate sensitivity by metabolizing in the mouth, tastants derived from food or saliva, or by the production of bioactive metabolites [182].

Two studies [183,184] have shown a strong correlation between taste perception, microbiota composition, and wine consumption. This conclusion is because polyphenols and oenological extracts (mainly red wine and grape seed byproducts) are effective antimicrobials against certain bacterial species. A study conducted by [159] compared the tongue microbiome of professional wine tasters and non-professional wine tasters and found that the microbiome of the first one, from the area further back of the tongue, had a higher abundance in specific bacterial genera such as *Streptococcus*, *Veillonella*, and *Prevotella*. On the other hand, this study also concluded that the microbiome of the dorsum of the tongue is influenced by the age and gender of the taster, and the frequency of wine-tasting sessions is negatively associated with microbiome diversity.

Besides all these factors, we must also consider human genetics and physiology. In some leading wine and wine-related products, phenolic compounds taste bitter. So, despite their healthy properties, many people do not like to eat plant-derived food, like vegetables, because of the bitterness associated with polyphenols [185]. Therefore, studying the sensory properties of polyphenol compounds is a prominent subject relevant to people’s food choices and the chemopreventive potential of food. It has been shown that hydrolyzable tannin and anthocyanins could be the primary compounds responsible for the bitterness of fruits and derived products, such as red wine [186]. Sweet and bitter transduction pathways have been discovered in parallel and share many mechanisms. In humans, bitter compounds bind to a variety of ~25 different receptors of the T2R family that can form both homomeric and heteromeric complexes [187]. Bitter perception is initiated by TAS2Rs, a family of G-protein-coupled receptors, codded by 25 bitter taste receptor family genes expressed on the surface of taste buds. The protein has an active site that is susceptible to the taste compound’s molecular structure [188]. However, different polyphenol compounds activate different combinations of bitter taste receptors, highlighting the complex relationships between bitter taste receptors, perception, evolution, and health. A reduction in selective pressure during human development has also been observed across the TAS2R family, possibly due to evolved changes in human biology and behavior [187,188].

The chemical structure of the taste compound can also influence its sensory perception. In general, amino acids (a.a) possess primary taste properties, in which aspartic acid and glutamic acid directly contribute to sour taste. These amino acids in the derived form of salt are umami, savory, and meaty. Meanwhile, L-amino acids with hydrophobic side chains, particularly leucine, isoleucine, tyrosine, and valine, are attributed to bitter taste [189,190]. D-amino acids, such as proline, alanine, lysine, glycine, serine, and threonine, are less commonly found than their more prevalent L-amino acid counterparts in nature, reflecting the prevailing predominance of L-amino acids in biochemical processes. Despite their relative scarcity, these D-form amino acids are recognized as having a sweet taste [191]. Thus, not only do natural sugars activate the sweet taste receptor T1R2/T1R3, but ligands with distinct chemical structures, such as amino acids and proteins, may bind to different domains of the sweet taste receptors [192]. The conformational change in the receptors upon ligand binding activates intracellular downstream signaling [193].

Regarding minerals, ions such as iron and copper may facilitate a retronasal perception of “metallic flavor perception,” defined as a combination of taste and retronasal odor [194]. This phenomenon occurs due to mineral salivary protein oxidation and the production of oxidation-related aldehydes related to odorant lipids.

## 6. Extraction Techniques of Nutraceutical Compounds in Wine and Wine-Related Products

Extraction is a process widely used in the food industry to increase the quality of food products. Since primordial times, long extraction periods and low efficiency led to the emergence of analytical methodologies [195,196].

The most basic and common technique for analyte extraction is solid–liquid extraction (SLE) or leaching. This unitary process allows the separation of substances in a solid matrix using organic solvents, forming a solution with the target analytes [197]. Soxhlet extraction (SOX) is an example of SLE, in which the solid sample is placed in a SOX thimble with an organic solvent. A condensation environment is created by heating under the reflux of the solvent. This exhaustive extraction takes about 12–24 h and uses large quantities of purified solvent in addition to the extensive time [16].

For liquid samples, ubiquitously used is liquid–liquid extraction (LLE), a process of transferring a dissolved substance from one liquid phase to another liquid phase. Usually, one phase is water, and the other is a non-polar organic solvent (immiscible or partially miscible solvent) [195].

As the complexity of products and food matrices to be analyzed has increased, there has been a growing demand for alternative extraction methods that are more efficient, faster, and more environmentally friendly with fewer solvents. These techniques can extract compounds from solid or liquid samples and are considered green extraction methods. There are several alternatives to conventional extraction methods. However, Table 3 shows the most applied extraction methods in wines and wine-related products [16,198].

Although some of these methods use organic solvents, compared to traditional methods, the amounts are reduced, and energy expenditure is lower than in conventional methods [16]. Other solutions exist beyond the alternative ways to extract health-promoting compounds in wine and wine-related products, such as green/sustainable solvents.

Ionic liquids (ILs) are salts that are liquid at or near room temperature due to mixing organic cations with organic or inorganic anions, i.e., a combination of molecules or at-oms electrified. Deep eutectic solvents (DESs) are another type of ionic liquid. Two or more substances make a eutectic mixture with lower melting points than any components. Natural eutectic solvents (NADESs) are a mixture naturally occurring in plants and animals. DESs and NADESs have high thermal stability and solubility and low toxicity [16,205].

After extracting nutraceutical compounds, purification and chemical characterization are common steps that can be taken to study further and develop the extracted compounds. The purification step removes unwanted impurities, and the chemical characterization involves identifying and quantifying the compounds extracted.

## 7. Separation and Identification of Health-Promoting Compounds

### 7.1. Chromatography Techniques

**Figure 5:**
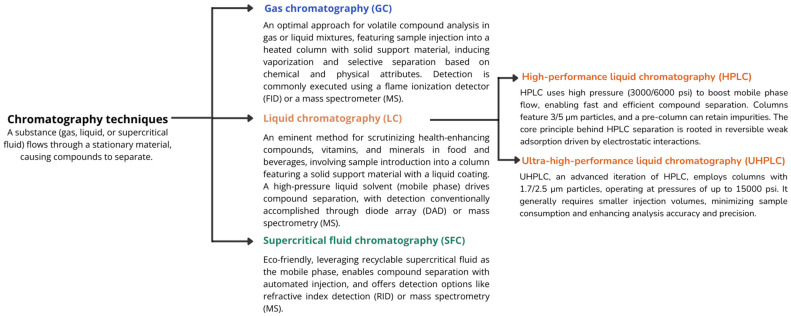
Overview of chromatographic techniques applied in analyzing health-promoting compounds in food and beverages (adapted from [196,206,207,208,210,211,212,213,214,215,216]).

Chromatography is a versatile and powerful technique capable of separating and identifying various compounds across various fields. Moreover, it offers valuable insights into the quality and quantity of individual components within a given sample [206,207,208,209]. While chromatographic methods may not provide precise compound identification, using internal standards, especially in techniques like high-performance liquid chromatography (HPLC), enhances accuracy by allowing for the comparison and verification of retention times or other parameters. This is particularly beneficial in discerning specific compounds with greater confidence. Incorporating hyphenated techniques in chromatography further contributes to the refinement of compound identification processes. Figure 5 summarizes metabolite analysis chromatographic techniques in food and beverages, defining each method and explaining their separation and detection mechanisms.

Recently, the study by Lukić et al. [217] highlights the utility of gas chromatography with time-of-flight mass spectrometric detection (TOF-MS) coupled with SPME (solid-phase microextraction) for identifying varietal markers of volatile compounds in wines made from non-aromatic red grapes. Besides that, this technique can contribute to maintaining the authenticity and quality of wines.

As a result, chromatograms with different peaks and retention times are obtained, which, through standards scrutinized under the same conditions as the samples, we can ascertain the compound eluted [206,207,208,211].

### 7.2. Spectroscopy Techniques

Spectroscopy techniques use the interaction of electromagnetic radiation with matter to study the properties or characteristics of materials. The interaction between the radiation and the material studied results in the radiation’s absorption, emission, or scattering, which can be used to obtain materials information about the material [218,219].

Table 4 provides a review of a range of spectroscopy techniques employed for the detection and measurement of health-promoting compounds present in wine and its related products. These spectroscopic methods hold significant significance in analyzing and evaluating advantageous constituents within these samples, thereby enhancing our comprehension and assessment of their potential health advantages.

Aleixandre-Tudo and Toit [220] wrote a chapter about the quantification of phenols during the winemaking process by UV-Vis spectroscopy, in which they refer that spectrophotometric methods are commonly used to analyze the content of total phenols, anthocyanins, tannins, and color, referring to several protocols that could be applied to wine and wine byproduct samples.

In 2017, Ríos-Reina et al. [222] utilized Attenuated Total Reflectance–Fourier-transform infrared spectroscopy (ATR-FTIR) to study the quality and authenticity of 67 wine vinegar samples registered as Protected Designation of Origin (PDO). ATR-FTIR, a spectroscopic technique that directly examines solid, liquid, or gel samples without elaborate preparation, proved instrumental in evaluating vinegar and authentically tracking the aging process.

### 7.3. Enzymatic Methods and Antioxidant Assays

In recent years, the biotechnology industry has grown due to the need for enzymatic and chemical tests for quick and easy detection of clinical cases [236]. Enzymatic methods use enzymes to catalyze chemical reactions of interest [237], which can be used as an extraction or direct technique for quantifying bioactive compounds [238]. Chemical tests are analytical techniques that involve using specific reagents or reactions to detect and identify the presence of particular compounds or elements in a sample [239,240].

**Figure 6:**
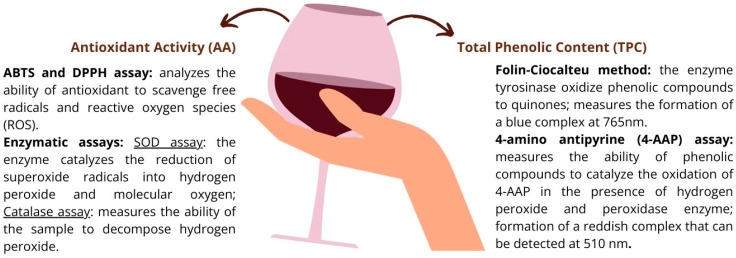
Main methods to quantify the total phenolic content (on the right) and antioxidant activity (on the left) in wine and wine-related products (adapted from [228,236,241,242,243,244]).

Figure 6 illustrates the ubiquitously used chemical and enzymatic methods for detecting and quantifying total phenolic content and measuring antioxidant activity in wines and wine-related products.

In a study with Greek red wines, Tekos et al. [245] evaluated the antioxidant profile of the samples using SOD, ABTS, and DPPH. The results showed high bioactivity in red wine, which may benefit human health.

Besides the previously mentioned methods for assessing antioxidant activity, two other assays commonly used are the FRAP (Ferric Reducing Antioxidant Power) assay and the ORAC (Oxygen Radical Absorbance Capacity) assay [246,247]. The FRAP assay determines the capacity of antioxidants in a sample to convert ferric ions (Fe^3+^) to ferrous ions (Fe^2+^) [248,249], while the ORAC assay measures the ability of the sample to inhibit peroxyl radicals and quantifies the extent of inhibition using a fluorescent probe [247,250].

### 7.4. Electrochemical Techniques

Electrochemical methodologies have emerged as alternatives to conventional and usual methods for determining nutraceutical compounds in food and beverages, namely amperometry and voltammetric methods [251,252]. These techniques are usually impregnated in biosensors to generate a more correct and quick response. Table 5 provides an overview of the prevalent electrochemical methods employed in the food industry to analyze health-promoting compounds.

Aiming to develop a biosensor to detect polyphenols in tea and wine, Datta et al. [253] used differential pulse voltammetry and electrochemical impedance spectroscopy (EIS) technology to characterize the samples, resulting in a sensitive and promising sensor.

**Table 5:** Compilation of the predominant electrochemical techniques employed in analyzing health-promoting compounds in beverages, encompassing wine and its related products.

Method	Method Description	References
**Amperometry**	Measures the current produced during an electrochemical reaction between the analyte and the electrode. The analyte is oxidized or reduced at the working electrode, and then the current is measured by a potentiostat since the analyte concentration is directly proportional to the current.	[251,252,254]
**Voltammetry**	Voltammetry measures the redox potential of chemical species, with cyclic voltammetry commonly used in the food industry. It involves cycling the voltage between two values at a specific scan rate on a working electrode, allowing oxidation and reduction reactions. The resulting voltammogram shows peaks and valleys corresponding to the behavior of electroactive species in the sample.	[228,251,252]
**Electrochemical impedance spectroscopy (EIS)**	Electrochemical impedance spectroscopy (EIS) measures electrical impedance changes in response to an applied voltage across various frequencies. It determines impedance by analyzing the ratio of the applied voltage to the resulting current.	[16,255]

## 8. Detection and Quantification of Health-Promoting Non-Phenolic Compounds

Following the extraction process, various techniques can be employed to identify and quantify non-phenolic compounds, including glutathione, vitamins, and minerals. Methods such as GC-MS, HPLC, voltammetry, and spectrometry can be valuable in investigating these compounds. These methodologies enable researchers to gain insights into the presence, concentration, and characteristics of these non-phenolic compounds in the samples under study [92,256,257].

Table 6 provides a comprehensive overview of alternative techniques frequently employed for analyzing glutathione, vitamins, and minerals in wines and wine-related products, which have yet to be previously reported in this study. The table summarizes the main methods used for identifying and quantifying these compounds, followed by the type and brief description of the technique.

## 9. Final Remarks

Understanding the composition of health-promoting compounds in wine and wine-related products is imperative for elucidating their potential impact on human health. This involves adept management of grapevine practices and mitigating biotic stresses, including temperature and nutrient considerations. The judicious selection of microorganisms for transforming grapes into wine, vinegar, or wine spirit allows for deliberately adjusting nutritional characteristics.

Wines primarily comprise phenolic and non-phenolic compounds that are pivotal in shaping consumers’ sensory perception and overall pleasantness. However, pH levels, enzymatic activity, and oral microbiome variations can significantly influence the nuanced perception of flavor and aroma. The detection and quantification of these health-promoting compounds demand a meticulous approach, considering factors such as the specificity of target compounds, sensitivity, selectivity, matrix complexity, cost implications, and responsiveness. Technological advancements, exemplified by sophisticated analytical methods like high-performance liquid chromatography (HPLC) and mass spectrometry (MS), have substantially elevated the precision and efficiency of compound analysis in wines, facilitating more accurate assessments of their nutritional and sensory attributes.

The practical implications of this knowledge lie in informing refined practices in viticulture and winemaking, addressing the discerning preferences of both connoisseurs and health-conscious consumers. This study lays the groundwork for continued exploration and refinement in the field, fostering a deeper understanding of the nexus between viticulture, winemaking, and human health. Advanced analytical techniques, such as those investigated in our global comparative studies, add a crucial layer to this research, enabling a comprehensive understanding of health-promoting compound variations across different geographic regions.

## Figures and Tables

**Table 1:** Prominent flavonoids and non-flavonoids identified in wine and its derivatives.

Group	Designation	Matrix
Flavonoids	Anthocyanins	Yeast-fermented beverages [39] Wine and grape [40]
	Flavonols	Rufete and Tempranillo wine [41] Flavored wine [42] Wine and grape [40]
	Flavanols	Rufete and Tempranillo wine [41] Wine and grape [40]
	Flavones	Flavored wine [42] Wine and grape [40]
	Flavanones	Wine [43] Wine and grape [40]
	Flavanes	Wine and grape [40]
	Flavononols	Wine and grape [40]
	Chalcones/ Dihydrochalcones	Wine and grape [40]
Non-flavonoids	Phenolic acids	Wine and grape [40]
	Tannins	Wine and grape [40]
	Stilbenes	Wine and grape [40,44]
	Coumarins	Wine and grape [40]
	Phenyl ethanol derivatives	Wine and grape [40]
	Lignans	Wine and grape [40] Wine [45]
	Neolignans	Wine and grape [40]

**Table 2:** Water-soluble vitamin contents (mg/L). Adapted from [92,93].

Group	Designation	Matrix	(mg/L) [92]	(mg/L) [93]
Vit. C	Ascorbic acid	Grape musts	30–572	
Vit. B1	Thiamine	Grape musts	0.08–1.20	0.89–3.51
Vit. B2	Riboflavin	Grape musts White Wine Red Wine	0.003–1.45 0.008–1.33 0.00047–0.0019	--
Vit. B3	Niacin	Grape musts Wines	0.79–8.80 0.11–0.42	0.02–3.24
Vit. B5	Pantothenic acid	Grape musts Grapes	0.00016–10−50 6.8–8.5	0.12–2.70
Vit. B6	Pyridoxine	Grape musts Red grapes White grapes	0.14–2.9 1.25 0.88	0.93–6.94
Vit. B7	Biotin	Red grape juices White grape juices Grape musts	0.00285 0.00147 0.0001–0.060	--
Vit. B8	Inositol	White wines Red wines	220–730 290–334	--
Vit. B9	Folic acid	Grape musts Wines	0.003–0.05 0.0004–0.0045	--

**Table 3:** Overview of the most applied extraction methods for studying wines and wine-related products.

Techniques	Solvent	Method Description	References
**SPME**	Solvent-free	A method based on the principle of adsorption/absorption and desorption through a silica fiber.	[199,200]
**UAE**	Organic solvent	Uses high-frequency ultrasonic waves to extract compounds due to high pressure and temperature.	[16,198,201]
**MAE**	Organic solvent	The sample is heated in a microwave oven, and the boiled solvent extracts the desired compounds.	
**SFE**	Supercritical fluids (SF) *	The sample, placed in a vessel, is submitted to an SF under high pressure and temperature that dissolves the target compound in the fluid. When the conditions decrease, the extract is collected.	[16,198,201]
**PLE** or **ASE**	Water or organic solvent	The compounds are extracted by applying controlled pressure and temperature to the solvent.	[16,198,201]
**PEF**	Solvent-free	A green, non-thermal method that uses electrodes and a solid electrical field to create stress in the cell membrane, leading to the formation of pores (irreversible or not).	[201,202]
**OH**	Solvent-free	An environmentally friendly alternative that combines electrical and thermal treatment to damage the cell membrane and increase the extraction of phenolic compounds.	[201,203]
**EAE**	Solvent-free	Enzymes allow the leaking of phenolic compounds or the recovery of these compounds from cell vacuoles, especially the insoluble-bound phenolics.	[16,204]

* Supercritical fluid (SF) is a substance (liquids or gases), commonly carbon dioxide, at temperature and pressure above its critical point and, at this point, has properties of both a gas and a liquid. Above the critical point, the substance has a density comparable to that of a liquid but with the diffusivity and compressibility of a gas [201]. SPME—solid-phase microextraction; UAE—ultrasound-assisted extraction; MAE—microwave-assisted extraction; SFE—supercritical fluid extraction; PLE—pressurized liquid extraction; ASE—accelerated solvent extraction; PEF—pulsed electric field; OH—ohmic heating; EAE—enzyme-assisted extraction.

**Table 4:** Review of spectroscopy techniques for identifying and quantifying health-promoting compounds in wine and wine-related products.

Technique	Method Description	References
**UV-Vis spectrophotometry**	Measures light absorption or transmission by a sample at specific wavelengths in the ultraviolet and visible regions (UV-Vis) of the electromagnetic spectrum (200–800 nm), usually following the Beer–Lambert law, which relates absorbance to concentration.	[219,220]
**Fourier-transform infrared spectroscopy (FTIR)**	Identifies chemical structure and functional groups in a compound by measuring their interaction with infrared light. FTIR instruments include transmission, reflection, and ATR (Attenuated Total Reflectance) types, each suited for different sample types. Transmission passes light through a thin sample, reflection reflects it off the sample’s surface, and ATR presses the sample against a diamond crystal.	[221,222]
**Nuclear magnetic resonance (NMR)**	Studying the properties of atomic nuclei in molecules, this analytical technique involves placing the sample in a strong magnetic field and applying a radiofrequency pulse. This process excites the nuclei, causing them to emit energy and produce a detectable signal in the spectroscope. The method is versatile, used for determining the structure of organic compounds, identifying substances through unique spectra, quantifying compound concentrations, and studying molecular dynamics in various scientific fields. Fourier-transform nuclear magnetic resonance (FT-NMR) is a common approach within this technique. It allows for more efficient data acquisition, improved spectral resolution, and enhanced sensitivity, making it a valuable tool for detailed molecular analysis.	[223,224,225,226]
**Raman spectroscopy**	Weigh the vibrational mode of molecules when a sample is illuminated with monochromatic light; a small portion of the scattered light shifts. This technique can be used to analyze samples in situ, without sample preparation or separation, and is highly selective because it can distinguish between different chemical species.	[227,228,229]
**Mass spectrometry**	Provides structural information about the separated compounds in combination with chromatographic techniques (hyphenated method). The compounds are ionized and passed through the equipment, which separates ions based on their mass-to-charge ratio (m/z), determining the target compounds’ molecular weight and chemical structure.	[230,231,232,233,234,235]

**Table 6:** Overview of alternative techniques for analyzing non-phenolic health-promoting compounds.

Technique	Type	Compounds	Description	References
**Acid titrations**	Chemical	Water-soluble vitamins	Acid-base (redox) titrations can be used to determine the content or concentration of certain vitamins. However, the specific method and procedure can vary depending on the analyzed vitamin.	[92]
**Atomic absorption spectrophotometer (AAS)**	Spectroscopic	Minerals (principally sodium, calcium, magnesium, and iron)	Analyze elements by measuring the absorption of light by atomized samples. It quantifies element concentration based on the amount of light absorbed by the atoms in the ground state. The sample is atomized using a flame or graphite furnace, and a specific wavelength of light is passed through it. The absorbed light is detected, and the absorbance corresponds to the element concentration in the sample.	[92,257,258,259,260]
**Differential pulse anodic stripping voltammetry (DPASV)**	Electrochemical	Minerals	A sensitive and selective method that analyzes trace metals and electroactive species in a sample. It involves the analyte accumulation on an electrode, followed by applying a voltage pulse to cause oxidation and stripping the analyte from the electrode surface. The resulting current peak is measured and used to determine the analyte concentration in the sample.	[258,259,261]
**Enzyme glutathione peroxidase assay**	Enzymatic	Glutathione	The enzyme catalyzes the reduction of hydrogen peroxide and other hydroperoxides in the presence of glutathione.	[262,263].
**Inductively coupled plasma–optical emission spectroscopy (ICP-OES)**	Spectroscopic	Minerals	This analytical method uses an inductively coupled plasma (high-temperature ionization source) to atomize and ionize the sample, then detect emitted light at specific wavelengths. It is handy for elemental analysis in a wide range of samples.	[257,261,264,265]
**Inductively coupled plasma–mass spectrometer (ICP-MS)**	Spectroscopic	Minerals	Employs an inductively coupled plasma as a high-temperature ionization source to atomize and ionize the sample. The ionized species are then introduced into a mass spectrometer, separated based on their m/z, and detected. This technique allows for the precise measurement of elemental and isotopic composition in a wide range of samples, offering high sensitivity and the ability to analyze multiple elements simultaneously.	[257,258,265,266,267]
**Microbiological assay (MA)**	Biological	Vitamins	These assays rely on the growth response of specific microorganisms (usually lactic acid bacteria), which require the vitamin to be analyzed for their growth and metabolism. The principle behind these assays is that the amount of change observed is directly proportional to the concentration of the vitamin in the sample.	[92]
**Thiochrome assay**	Chemical	Vitamin B1	Thiamine is converted to thiochrome in the presence of a thiol reagent and an oxidizing agent. The thiochrome formed exhibits fluorescence, which can be measured and correlated with the thiamine concentration in the sample.	[92,258]

## Data Availability

No new data were created or analyzed in this study. Data sharing does not apply to this article.
